# Safety and immunogenicity of a randomized phase I prime-boost trial with ALVAC-HIV (vCP205) and gp160 MN/LAI-2 adjuvanted in alum or polyphosphazene

**DOI:** 10.1186/1742-4690-9-S2-O50

**Published:** 2012-09-13

**Authors:** RJ O'Connell, VR Polonis, S Ratto-Kim, J Cox, LL Jagodzinski, J Malia, NL Michael, J Excler, ML Robb, JH Kim

**Affiliations:** 1 U.S.Military HIV Research Program, Bethesda, MD, USA; 2 International AIDS Vaccine Initiative, New York, NY, USA

## Background

ALVAC-HIV prime/HIV-1 Env protein boost regimens have shown HIV-specific neutralizing antibody (NAb) and cell-mediated immune responses, but the impact of protein subunit schedule and adjuvant requires further definition.

## Methods

A Phase 1 trial was conducted in two parts. In Part A, (open-label) 19 volunteers received oligomeric gp160 MN/LAI-2 (ogp160) with a dose escalation (25, 50, 100 μg). In Part B, 72 volunteers (60 active, 12 placebo) received placebo or recombinant canarypox expressing HIV-1 antigens, (ALVAC-HIV, vCP205) prime with different doses and schedules of ogp160MN/LAI-2 in alum or polyphosphazene (PCPP).

## Results

The vaccines were safe and well tolerated with no vaccine-related serious adverse events. Cumulative chromium release CTL frequency was 37%, and 54% of volunteers showed proliferative responses to HIV antigens. Lymphoproliferative CD4+, HIV-specific responses were seen in 53% of ogp160 only and 57% of prime-boost recipients, respectively. Induced binding antibody to ogp160 was dose-dependent. NAb responses to vaccine homologous Tier 1 HIV-1 MN were seen in 99% of vaccine recipients. While NAb to the heterologous Tier 2 US-1 (R5, clade B) pseudovirus was negative in all volunteers tested using TZM-bl cells, in a PBMC-based assay, US-1 primary isolate Nab was induced in 2/19 (10.5%) recipients of ogp160 protein alone and in 5/30 (16.7%) prime-boost volunteers who received ogp160 in PCPP. Primary isolate neutralization was observed more frequently overall in recipients of ogp160 in PCPP, as compared with alum (p=0.027). Using an intracellular p24 flow-cytometry assay, sera from an ALVAC-HIV/ogp160 recipient demonstrated 94% neutralization of US-1.

## Conclusion

A small percentage of vaccine recipients developed Nab to heterologous primary isolates, responses that to our knowledge have not been previously described. These results constitute proof of concept that Tier 2 NAb can be elicited by vaccination in humans, and underscore the importance of further optimization of prime-boost vaccination and adjuvanting strategies for HIV-1 prevention.

